# CILP-2 is a novel secreted protein and associated with insulin resistance

**DOI:** 10.1093/jmcb/mjz016

**Published:** 2019-06-06

**Authors:** Tong Wu, Qin Zhang, Shaobo Wu, Wenjing Hu, Tingting Zhou, Ke Li, Dongfang Liu, Harvest F Gu, Hongting Zheng, Zhiming Zhu, Ling Li, Gangyi Yang

**Affiliations:** 1 Department of Endocrinology, The Second Affiliated Hospital, Chongqing Medical University, Chongqing Clinical Research Center for Geriatrics, Chongqing, China; 2 Key Laboratory of Diagnostic Medicine (Ministry of Education) and Department of Clinical Biochemistry, College of Laboratory Medicine, Chongqing Medical University, Chongqing, China; 3 Center for Pathophysiology, School of Basic Medicine and Clinical Pharmacy, China Pharmaceutical University, Nanjing, China; 4 Department of Endocrinology, Xinqiao Hospital, Third Military Medical University, Chongqing, China; 5 Department of Hypertension and Endocrinology, Daping Hospital, Third Military Medical University, Chongqing Institute of Hypertension, Chongqing, China

**Keywords:** CILP-2, secreted protein, insulin resistance, type 2 diabetes

## Abstract

Genetic association studies have implicated that cartilage intermediate layer protein 2 (CILP-2) confers the risk susceptibility for type 2 diabetes (T2DM). However, it is still unknown whether CILP-2 is involved in the regulation of glucose homeostasis and insulin resistance (IR). In the current study, we initially observed that CILP-2 as a secreted protein was detected in both conditioned medium and lysates of cells transfected with an overexpressed vector. We then found that circulating CILP-2 levels had a progressive increase from normal to impaired glucose tolerance (a pre-diabetic status) and then to diabetes, which was correlated positively with waist-to-hip ratio, triglyceride, fasting blood glucose, 2-h blood glucose after glucose overload, HbA1c, fasting insulin, 2-h plasma insulin after glucose overload, and homeostasis model assessment of insulin resistance but negatively with HDL-C. CILP-2 expression was increased in the liver and muscle but decreased in adipose tissues of obese mice or T2DM patients. Furthermore, we demonstrated that CILP-2 circulating levels were affected by OGTT and Exenatide. CILP-2 overexpression resulted in impaired glucose tolerance and hepatic IR *in vivo* and increased PEPCK expression whereas suppressed phosphorylation of insulin receptor and Akt kinase *in vitro*. Based on these findings, we have identified a direct interaction between CILP-2 and PEPCK and suggested that CILP-2 plays an important role in the regulation of hepatic glucose production.

## Introduction

Cartilage intermediate layer protein 2 (CILP-2) is a cartilage intermediate layer protein and expressed most abundantly in cartilaginous tissues (Bernardo et al., 2011). As a glycoprotein, it may contribute to the structural organization and matrix architecture within these connective tissues (Willer et al., 2008; Tai et al., 2009; Zhou et al., 2011; Saxena et al., 2012; Jia et al., 2014). Furthermore, CILP-2 is presented in skeletal muscle and may play the roles in extracellular matrix structure and function of non-cartilaginous tissues (Bernardo et al., 2011).

Several genetic association studies have demonstrated that CILP-2 genetic polymorphisms are associated with low-density lipoprotein cholesterol (LDL-C), triglyceride (TG) levels, and coronary heart disease risk in European population (Willer et al., 2008), high-density lipoprotein cholesterol (HDL-C) levels in Malaysian population (Tai et al., 2009), and LDL-C and HDL-C levels in Chinese population (Zhou et al., 2011). Furthermore, a meta-analysis of 39 multiethnic type 2 diabetes mellitus (T2DM) association studies has implicated that CILP-2 confers the risk susceptibility for T2DM (Saxena et al., 2012). However, whether CILP-2 plays a role in the regulation of glucose homeostasis and insulin resistance (IR) is unknown. The aim of the current study is to investigate the influence of CILP-2 on insulin sensitivity and the mechanism beneath.

In the current study, we first identified CILP-2 as a novel secreted protein in human blood. We then analyzed the circulating CILP-2 levels in healthy and impaired glucose tolerance (IGT) individuals as well as newly diagnosed T2DM patients (nT2DM). We also examined CILP-2 expression in fat and muscles from T2DM patients and in insulin targeted tissues from animal models of IR. Furthermore, we evaluated whether the circulating CILP-2 levels were impacted by overnight fast (a low-insulin and hypoglycemia condition), oral glucose challenges (a hyperglycemic and hyperinsulinemic condition), euglycemic-hyperinsulinemic clamp (EHC, euglycemic-hyperinsulinemic condition), and anti-diabetic agent (Exenatide, a natural analogue of GLP-1) in T2DM patients or healthy individuals. Finally, we evaluated the impacts of CILP-2 overexpression on hepatic glucose fluxes and insulin signaling *in vivo* and *in vitro* and identified a direct interaction between CILP-2 and phosphoenolpyruvate carboxykinase (PEPCK) in the regulation of hepatic glucose production (HGP).

## Results

### CILP-2 is a secreted protein and presents in human serum

**Figure 1:**
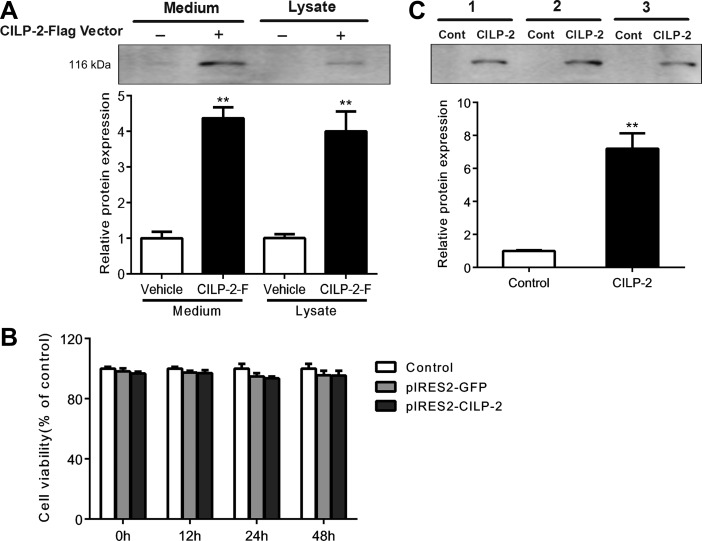
CILP-2 is identified as a secreted protein. (**A**) Immunoblots of CILP-2 protein from conditioned medium and cell lysates of HEK-293T cells transfected with pIRES2-EGFP-CILP-2-Flag (CILP-2-F) (+) or an empty vector (–). (**B**) Transfection of CILP-2-F in HEK-293T cells did not affect the cell viability as determined with CCK-8 test. (**C**) Detection of CILP-2 in human serum. Serum (500 μl) from 3 individuals was separately immunoprecipitated with CILP-2 antibody (CILP-2) or pre-immune IgG (Control). **P < 0.01 compared with vehicle or control.

We first overexpressed pIRES2-EGFP-CILP-2-Flag (CILP-2-F) cDNA in HEK-293T cells. As shown in Figure 1A, CILP-2-F was visible in conditioned medium and cell lysates treated with CILP-2-F. Cell count kit-8 colorimetric assay (CCK-8) experiment showed that the transfection of CILP-2-F did not affect the cell viability (Figure 1B), indicating that increased CILP-2 protein expression in conditioned medium was due to increased secretion rather than release by cell damage. The data indicated that CILP-2 was a secreted protein. In serum samples from three subjects, we found a band corresponding to CILP-2 in the lanes. The lanes were incubated with CILP-2 antibody but not with control serum, suggesting that CILP-2 is a protein in circulation (Figure 1C).

### Circulating CILP-2 and its associations with anthropometric and biochemical parameters

**Figure 2:**
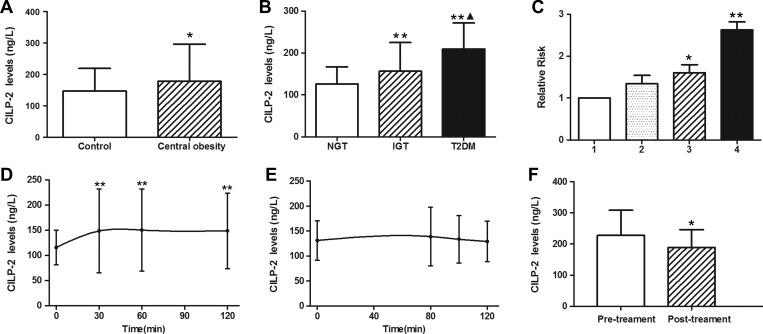
Circulating CILP-2 levels in study population. (**A**) Circulating CILP-2 levels according to waist circumference (WC, central obesity: male ≥ 94 cm; female ≥ 80 cm). (**B**) Circulating CILP-2 levels in NGT, IGT, and T2DM. (**C**) Prevalence of elevated T2DM in different quartiles of CILP-2 (Quartile 1, <119.4 ng/L; Quartile 2, 119.4–136.7 ng/L; Quartile 3, 136.7–165.2 ng/L; Quartile 4, >165.2 ng/L). (**D**) Circulating CILP-2 levels in healthy subjects during OGTT. (**E**) Circulating CILP-2 levels in healthy subjects during EHC. (**F**) Circulating CILP-2 levels in T2DM patients treated with Exenatide. *P < 0.05, **P < 0.01 vs. controls, NGT, Quartile 1, 0 min, or pretreatment. NGT, normal controls; IGT, impaired glucose tolerance; T2DM, type 2 diabetes mellitus. ^▴^*P* < 0.05 vs. IGT. Data are expressed as mean ± SD.

In healthy subjects, circulating CILP-2 levels were ranged from 63.9 to 351.8 ng/L and no significant difference between males and females was observed. However, the subjects with central obesity (*n* = 163), defined by waist circumference (WC, male ≥ 94 cm and female ≥ 80 cm) (Methot et al., 2010), had significantly higher circulating CILP-2 levels than those without central obesity (Figure 2A). Significantly higher circulating CILP-2 levels were observed in IGT subjects and nT2DM patients compared to controls (Table 1) and also in nT2DM patients compared with IGT subjects (*P* < 0.01, Figure 2B). In addition, circulating CILP-2 levels were correlated positively with waist-to-hip ratio (WHR), TG, fasting blood glucose (FBG), 2-h blood glucose after glucose overload (2h-BG), HbA1c, fasting insulin (FIns), 2-h plasma insulin after glucose overload (2h-Ins), and homeostasis model assessment of insulin resistance (HOMA-IR) but negatively with HDL-C (Supplementary Table S1). Multivariate regression analyses showed that WHR and HOMA-IR were independently related factors influencing circulating CILP-2 levels (Supplementary Table S1). Binary logistic regression analyses showed that circulating CILP-2 was significantly associated with IGT and T2DM even after controlling for anthropometric variables and lipid profile (Supplementary Table S2). Furthermore, the relative risks for T2DM were increased in parallel with increasing CILP-2 quartiles (Figure 2C).

**Table 1:** Clinical characteristics and circulating CILP-2 levels in study subjects.

Item	NGT (*n* = 105)	IGT (*n* = 102)	T2DM (*n* = 103)
Age (year)	52.3 ± 9.6	54.1 ± 10.8	55.0 ± 11.5
Males/females	36/69	41/61	41/62
BMI (kg/m^2^)	23.7 ± 3.3	24.0 ± 3.1	24.7 ± 3.1^*^
WHR	0.87 ± 0.06	0.90 ± 0.05^**^	0.91 ± 0.06^**^
FAT (%)	30.40 ± 8.46	30.79 ± 6.70	30.88 ± 8.40
SBP (mmHg)	119.1 ± 16.9	123.4 ± 16.2^*^	127.1 ± 16.3^**^
DBP (mmHg)	74.3 ± 10.5	77.1 ± 9.15^*^	79.2 ± 9.6^**^
TG (mmol/L)	1.23 (0.90–1.81)	1.33 (0.98–1.93)	1.75 (1.24–2.27)^**,††^
TC (mmol/L)	4.84 ± 1.19	5.06 ± 1.07	5.15 ± 0.98^*^
HDL-C (mmol/L)	1.33 ± 0.30	1.29 ± 0.32	1.28 ± 0.26
LDL-C (mmol/L)	3.00 ± 0.80	3.00 ± 0.88	3.06 ± 1.08
FFA (umol/L)	0.54 ± 0.21	0.54 ± 0.30	0.61 ± 0.26^*,†^
FBG (mmol/L)	5.00 ± 0.61	5.77 ± 0.75^**^	9.16 ± 4.35^**,††^
2h-BG (mmol/L)	6.12 ± 1.13	9.19 ± 0.98^**^	17.52 ± 7.13^**,††^
FIns (mU/L)	6.90 (5.10–9.60)	8.69 (6.10–12.49)^**^	9.20 (5.95–13.17)^**^
2h-Ins (mU/L)	36.5 (17.0–54.5)	70.5 (54.2–112.9)^**^	52.9 (23.1–82.4)^**,††^
HbA1c (%)	5.74 ± 0.55	5.95 ± 0.48^**^	7.92 ± 2.40^**,††^
HOMA-IR	1.75 ± 0.95	2.68 ± 1.70^**^	3.97 ± 2.41^**,††^
HOMA-IS	96.2 (55.9–147.6)	80.1 (59.7–127.4)	46.3 (21.7–68.8)^**,††^
CILP-2 (ng/L)	126.1 ± 40.7	156.7 ± 68.4^**^	209.4 ± 142.2^**,††^
CILP-2 (adjusted)^§^	126.4 ± 9.4	156.1 ± 9.9^**^	209.0 ± 9.6^**,††^

Values are given as mean ± SD or median (interquartile range). ^§^Mean ± standard error by general linear model with adjustment of age, gender, and BMI. ^*^P < 0.05, ^**^P < 0.01 compared with NGT group; ^†^*P* < 0.05, ^††^*P* < 0.01 compared with IGT group.

### The effects of hyperglycemia, hyperinsulinemia, and Exenatide treatment on circulating CILP-2

To validate whether CILP-2 was impacted by the elevation of blood glucose and insulin, circulating CILP-2 levels were analyzed at different time points during oral glucose tolerance test (OGTT) and EHC in normal individuals. As depicted in Figure 2D, after oral glucose uptake, circulating CILP-2 levels were significantly and rapidly increased from 115.7 ± 34.3 to 148.7 ± 74.9 ng/L at 2 h (all *P* < 0.01 vs. 0 h). During the EHC, circulating CILP-2 levels had a slight non-statistically significant increase (Figure 2E). Finally, we evaluated whether Exenatide treatment alters CILP-2 levels in T2DM patients. After 12 weeks of Exenatide treatment, free fatty acids (FFA), FBG, postprandial blood glucose (PBG), and HOMA-IR in T2DM patients had significantly decreased (Supplementary Table S3). Interestingly, circulating CILP-2 levels were also significantly decreased with Exenatide treatment (vs. pre-treatment, *P* < 0.05, Figure 2F).

### CILP-2 mRNA and protein expression in mouse and human tissues

**Figure 3:**
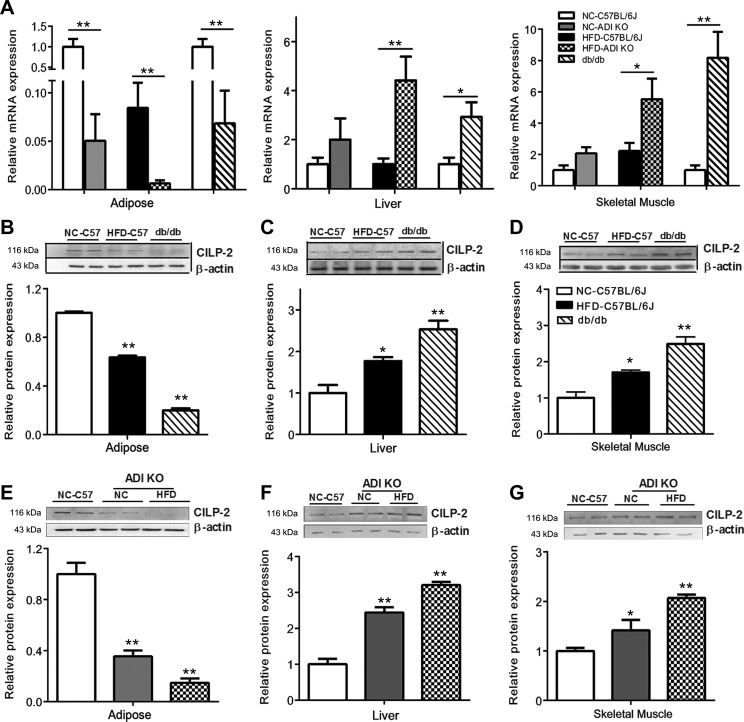
CILP-2 expression in mice. (**A**) CILP-2 mRNA expression in the liver, adipose, and muscle tissues of NC- or HFD-fed C57BL/6 J, ADI KO, and db/db mice. (**B–D**) CILP-2 protein expression in the adipose tissue (**B**), liver (**C**),and skeletal muscle (**D**) of NC- or HFD-fed C57BL/6 J and db/db mice. (**E–G**) CILP-2 protein expression in the adipose tissue (**E**), liver (**F**), and skeletal muscle (**G**) of NC-fed C57BL/6 J mice and NC- or HFD-fed ADI KO mice. NC, standard chow diet; HFD, high-fat diet; ADI, adiponectin. Data are expressed as mean ± SEM. *P < 0.05, **P < 0.01 compared with NC- or HFD-fed C57BL/6 J mice.

**Figure 4:**
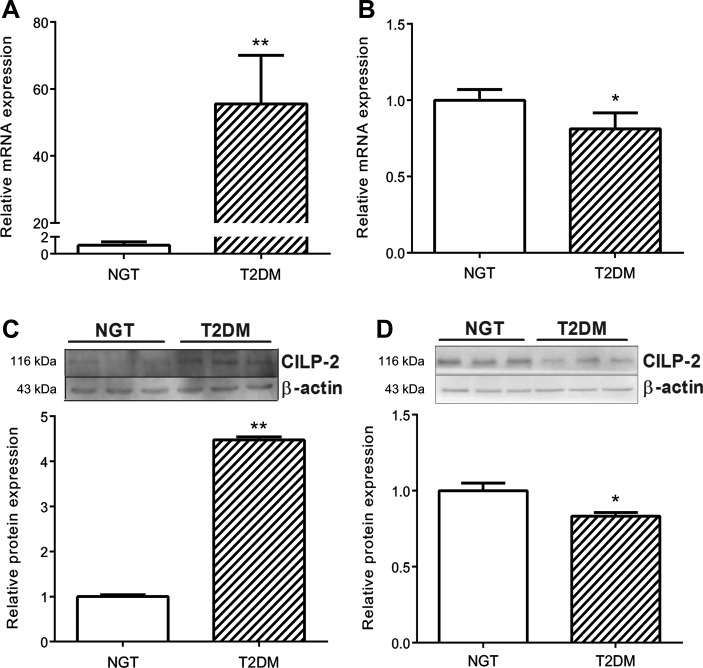
CILP-2 mRNA and protein expression in muscle and adipose tissues from healthy and T2DM subjects. (**A** and **B**) CILP-2 mRNA expression in the skeletal muscle (**A**) and adipose tissue (**B**). (**C** and **D**) CILP-2 protein levels in the skeletal muscle (**C**) and adipose tissue (**D**). Data are presented as mean ± SEM (*n* = 10). *P < 0.05, **P < 0.01 relative to NGT subjects.

We examined the expression of CILP-2 in different tissues of mice. The results showed that CILP-2 was mainly expressed in muscle, including myocardium, fat and liver tissues (Supplementary Figure S1A and B). Therefore, we considered the major resources of CILP-2 in blood were from these tissues. We further analyzed the expression patterns of CILP-2 in mice and human with and without IR. We found that in adipose tissues, mRNA and protein expressions of CILP-2 were significantly decreased in high-fat diet (HFD)-fed C57BL/6 J (WT) and standard chow diet (NC)- or HFD-fed adiponectin knockout (ADI KO) mice, and db/db mice compared with NC-fed WT mice (*P* < 0.05 or *P* < 0.01, Figure 3A, B, and E). In the liver and muscle, the expressions of CILP-2 mRNA and/or protein were markedly increased in NC-fed ADI KO mice, HFD-fed ADI KO mice, and db/db mice compared to NC-fed WT mice (*P* < 0.05 or *P* < 0.01, Figure 3A, C, D, F, and G). To investigate whether CILP-2 expression was altered in T2DM, we applied Quantitative Real-Time PCR (qRT-PCR) and Western blots for analysis in skeletal muscle and adipose tissues obtained from healthy subjects and T2DM patients (Supplementary Table S4). The results showed that CILP-2 was expressed in human muscle and fat tissues. When compared with controls, CILP-2 mRNA expression and protein levels were markedly increased in muscle tissues in T2DM patients (Figure 4A and C), whereas CILP-2 expression was reduced in the fat of T2DM individuals (Figure 4B and D).

### Effects of CILP-2 overexpression in the liver on glucose homeostasis and insulin sensitivity in vivo

**Figure 5:**
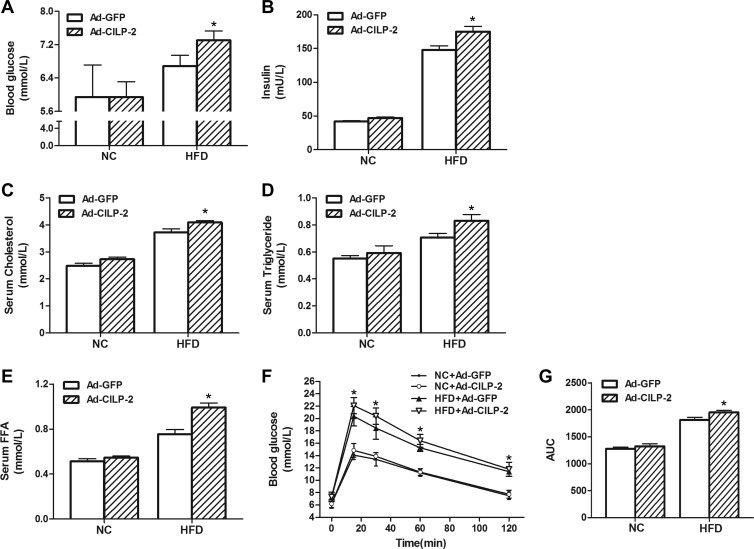
The effects of CILP-2 overexpression on metabolic parameters *in vivo*. (**A**) Fasting blood glucose. (**B**) Fasting insulin. (**C**) Serum cholesterol. (**D**) Serum triglyceride. (**E**) Serum free fatty acid. (**F**) Blood glucose during OGTT. (**G**) The AUC for glucose during OGTT. NC, standard chow diet; HFD, high-fat diet. Values are mean ± SEM (*n* = 5). *P < 0.05 vs. Ad-*GFP*.

We injected WT mice with recombinant adenovirus vectors containing CILP-2 gene (Ad-*CILP-2*) or recombinant adenovirus encoding enhanced green fluorescence protein (Ad-*GFP*) via tail-vein and found that the hepatic mRNA and protein expressions of CILP-2 were markedly increased in liver of Ad-*CILP-2* mice (Supplementary Figure S2). Furthermore, the circulating CILP-2 levels were also significantly increased after Ad-*CILP-2* injection in Ad-*CILP-2* mice (Supplementary Figure S3). Increased CILP-2 expression significantly increased FBG, insulin, TC, TG, and FFA levels in HFD-fed mice (Figure 5A–E). When fed an HFD, intraperitoneal injection of a bolus of glucose increased blood glucose levels to a larger extent in Ad-*CILP-2* mice than in Ad-*GFP* littermates (Figure 5F). The area under the curve (AUC) for glucose was also significantly increased in Ad-*CILP-2* mice compared with Ad-*GFP* mice (Figure 5G).

**Figure 6:**
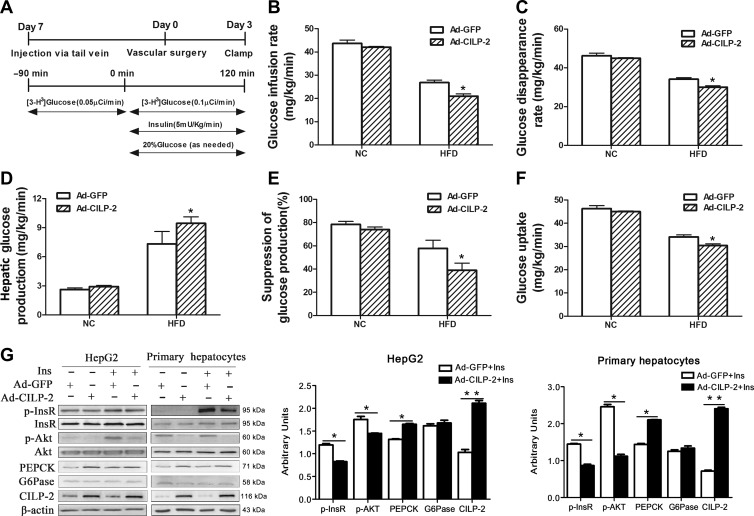
Overexpression of CILP-2 using Ad-*CILP-2* exacerbates insulin resistance *in vivo* and *in vitro.* (**A**) Experimental protocol for Ad-*CILP-2* delivery and insertion of catheters and clamp protocol. (**B**) Glucose infusion rate (GIR). (**C**) Glucose disappearance rates (GRd). (**D**) Hepatic glucose production (HGP). (**E**) Percentage of the suppression of endogenous glucose production. (**F**) Whole-body glucose uptake. (**G**) HepG2 cells and MPHs were treated as indicated in Materials and methods. Western blots (left) and quantitative measurements of p-InsR, p-Akt, PEPCK, G6Pase, and CILP-2 protein (right). NC, standard chow diet; HFD, high-fat diet; MPH, mouse primary hepatocytes. Values are mean ± SEM (*n* = 5 for *in vivo* study). *P < 0.05, **P < 0.01 vs. Ad-*GFP*.

To further assess the role of CILP-2 in the regulation of insulin sensitivity, EHCs were performed on mice fed HFD or NC (Figure 6A). As shown in Figure 6B and C, when fed an HFD, the glucose infusion rate (GIR) and glucose disposal rate (GRd) were decreased ~24% and 12%, respectively, in Ad-*CILP-2* mice than in Ad-*GFP* mice (*P* < 0.05). These results indicated that CILP-2 overexpression further decreased whole-body insulin sensitivity in HFD-fed mice. To examine the impact of CILP-2 overexpression on hepatic insulin sensitivity, hepatic glucose kinetics was assessed by tracer dilution methodology. When fed with HFD, the suppression of HGP by hyperinsulinemia was 73% in Ad-*GFP* mice, but only ~48% in Ad-*CILP-2* mice relative to NC-fed Ad-*GFP* mice (*P* < 0.05, Figure 6D and E), indicating a marked decrease in hepatic insulin sensitivity. In HFD-fed Ad-*CILP-2* mice, peripheral glucose uptake was also slightly decreased compared with HFD***-***fed Ad-*GFP* mice (Figure 6F).

### CILP-2 overexpression in the liver suppresses insulin-stimulated phosphorylation of InsR and Akt in vitro

Based on the results described above, we speculated that CILP-2 might be involved in the regulation of hepatic gluconeogenesis and insulin signaling. To test this hypothesis, HepG2 and Mouse primary hepatocytes (MPHs) were transfected with Ad-*CILP-2* or Ad-*GFP* followed stimulation with or without insulin. As predicted, CILP-2 expression at protein levels was significantly increased in Ad-*CILP-2* treated cells (Figure 6G). In addition, CILP-2 protein secretion in cell medium was also significantly increased (Supplementary Figure S4). Consistent with the results of *in vivo* study, the protein expression of PEPCK, a key gluconeogenic enzyme, was significantly increased in Ad-*CILP-2* treated HepG2 and MPHs compared with the controls. Importantly, insulin-stimulated phosphorylation of insulin receptor (InsR) and Akt kinase (Akt) was found to be significantly decreased in Ad-*CILP-2* treated HepG2 and MPHs (Figure 6G).

### Effects of CILP-2 overexpression in the muscle on glucose homeostasis and insulin sensitivity

**Figure 7:**
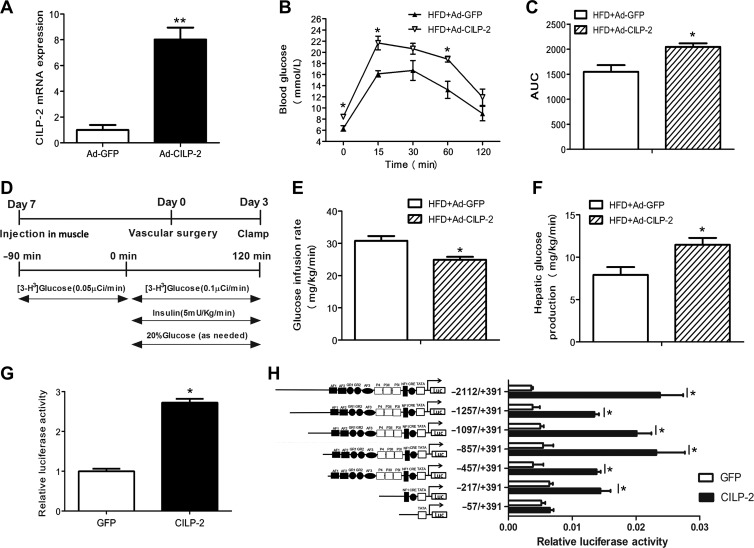
CILP-2 affects glucose metabolism and activates the PEPCK promoter. (**A**–**F**) HFD-fed C57BL/6 J mice were injected with Ad-*CILP-2* or Ad-*GFP* as indicated in Materials and methods. (**A**) The expression of CILP-2 mRNA in muscle tissues. (**B**) Blood glucose during GTT. (**C**) The AUC for glucose during GTT. (**D**) Experimental protocol for Ad-*CILP-2* delivery, insertion of catheters, and the EHC. (**E**) Glucose infusion rate (GIR). (**F**) Hepatic glucose production. HFD, high-fat diet. (**G** and **H**) HEK293 cells were treated as indicated in Materials and methods. (**G**) CILP-2 induced PEPCK promoter activity in HEK293 Cells. (**H**) Identification of CILP-2 action region in the PEPCK promoter by deletion analysis. Values are mean ± SEM (*n* = 5 for *in vivo* study). *P < 0.05, **P < 0.01 vs. Ad-*GFP* or GFP.

Because CILP-2 is also secreted by muscle tissues, we injected HFD-fed WT mice with Ad-*CILP-2* or Ad-*GFP* by bilateral gastrocnemius muscles and found that the expression of CILP-2 mRNA was markedly increased in the muscle of Ad-*CILP-2* mice (Figure 7A). During GTTs, blood glucose levels were increased to a larger extent in Ad-*CILP-2* mice than in Ad-*GFP* littermates (Figure 7B). AUC was also significantly increased in Ad-*CILP-2* mice compared with Ad-*GFP* mice (Figure 7C).

To further evaluate the role of CILP-2 secreted by muscle tissues in glycoregulation, EHCs were performed on mice fed with HFD (Figure 7D). In Ad-*CILP-2* mice, GIR was decreased ~20%, while HGP was increased ~32% compared with those of Ad-*GFP* mice (Figure 7E and F). These results indicate a marked reduction in insulin sensitivity.

### High glucose increases CILP-2 mRNA expression and secretion and CILP-2 increases PEPCK promoter activity

To further evaluate the relationship between glucose and CILP-2, we observed the influence of high glucose on CILP-2 expression and secretion in HepG2 cells. As shown in Supplementary Figure S5, the mRNA expression of CILP-2 in HepG2 cells was significantly increased under high glucose condition. In addition, high glucose also led to increased CILP-2 concentration in culture media. These results suggested that the glucose might regulate with the expression and secretion of CILP-2.

Because Ad-*CILP-2* increased the expression of PEPCK in hepatocytes, which increases HGP *in vivo*, we speculated that CLIP-2 may have an interaction with PEPCK gene transcription in hepatocytes. To determine whether CILP-2 influences PEPCK at the transcriptional level, the impact of CILP-2 on PEPCK transcription was observed in HEK-293 cells by luciferase reporter assay. As shown in Figure 7G, PEPCK promoter activity was higher in the HEK-293 cells treated with CILP-2-F, compared to those transfected with pIRES2-EGFP.

To define the promoter region responsible for the CILP-2 regulation of PEPCK expression, we constructed various 5′ deletions of the PEPCK promoter plasmid, pGL3-PEPCK (–2112/+391)-luc. The dual luciferase assay showed that the transcriptional activity of CILP-2 to regulate PEPCK promoter was significantly increased in pGL3-PEPCK (–2112), pGL3-PEPCK (–1257), pGL3-PEPCK (–1097), pGL3-PEPCK (–857), pGL3-PEPCK (–457), and pGL3-PEPCK (–217), but not in pGL3-PEPCK (–57) (Figure 7H). This result suggested that a potential CILP-2-regulating site of the PEPCK promoter may be located in –217 bp to –57 bp upstream of the transcriptional start site, which is the site of NF1 or CRE.

## Discussion

Identification of novel secreted proteins or cytokines in circulation contributing to the development of IR is of importance in basic research and clinical observation. In the current study, we first screened the complete CILP-2 protein sequence in humans from the Swiss-Prot database (SWISS PROT accession no: Q8IUL8) to investigate whether CILP-2 may be a secretory protein. The results showed that most of the 5′ methionine residue of CILP-2 is immediately followed by a typical signal peptide sequence as predicted according to the method described previously (Nielsen et al., 1997; Bendtsen et al., 2004; Hiller et al., 2004). The predicted cleavage site for the signal peptide lies between the alanine and arginine acids at positions 20 and 21, respectively. Therefore, we provided evidence suggesting that CILP-2 may be a secreted protein. We then demonstrated that CILP-2 is detectable in culture media from HEK-293T cells transfected with CILP-2-F to further confirm that CILP-2 is a secreted protein. At the same time, the cell viability indicated by CCK-8 test is not impacted by plasmid transfection. Thereby, the data implicate that increased CILP-2 levels in culture medium are due to increased secretion rather than release due to cell destruction. Furthermore, in human serum, an abundance of CILP-2 protein is detected. The results have provided the positive evidence that CILP-2 is a novel secreted protein in human blood. We further examined circulating CILP-2 levels in human and demonstrated that circulating CILP-2 levels were markedly increased in central obesity, IGT and nT2DM subjects compared with healthy controls. Notably, circulating CILP-2 levels in the studied population were gradually elevated from normal subjects to IGT (pre-diabetes) and to T2DM, following increased blood glucose levels. We also found that circulating CILP-2 was correlated significantly with parameters of adiposity (WHR), dyslipidemia (TG and HDL-C), blood glucose and IR (HOMA-IR). These results reveal an alteration in circulating CILP-2 over time from a pre-diabetic to a diabetic state and suggest that CILP-2 is involved in the development of dysmetabolism and IR. However, the underlying mechanism concerning increased circulating CILP-2 levels in IR individuals still remained unknown. We speculated that elevated circulating CILP-2 levels might be due to the impairment of CILP-2 signaling in target tissues and/or the dysregulation of CILP-2, which is caused by changes in biosynthesis and release or in response to hyperglycemia, hyperinsulinemia or adipokines in IR state.

To test the speculation, we assessed the effect of an oral glucose challenge on circulating CILP-2 in healthy subjects. A notable finding a significant increase circulating CILP-2 levels until 2-h after an oral glucose challenge. This pattern is different from those of insulin and glucose during OGTT. The results of OGTT and positive correlation of HbA1c and HOMA-IR with circulating CILP-2 implicated an effect of hyperglycemia or hyperinsulinemia on CILP-2 release.

As the increase in glucose after an oral glucose challenge is accompanied by an increase in insulin levels, it is unclear whether short-term hyperglycemia or hyperinsulinemia is responsible for the CILP-2 release. To address this question, we further performed EHCs to control blood glucose levels and investigated the impact of hyperinsulinemia alone on CILP-2 levels in healthy individuals. Our results indicate that acute hyperinsulinemia failed to lead to changes of circulating CILP-2 levels. In the *in vitro* study, we also found that high glucose stimulated the expression and secretion of CILP-2 in HepG2 cells. Therefore, hyperglycemia may stimulate the secretion and release of CILP-2, as hyperglycemia promotes insulin release.

Oral glucose ingestion stimulates the incretin release, while incretin release is represented by a postprandial elevation of GLP-1 (Creutzfeldt, 1992). Moreover, GLP-1 may activate or inhibit some cytokine release, such as adiponectin and visfatin (Li et al., 2008, 2011a). Therefore, it is important to investigate the effect of GLP-1 on circulating CILP-2 *in vivo*. In an interventional study, we evaluated whether Exenatide, a GLP-1 analogue, would affect circulating CILP-2 levels in T2DM patients. After 12 weeks of Exenatide administration, we observed a significant descent in circulating CILP-2 levels, following improved glycemic control and insulin sensitivity. Based upon this finding, we speculate that the effect of Exenatide treatment on circulating CILP-2 may be due to a result of metabolic changes. However, future studies are needed to evaluate the direct effect of GLP-1 analogue on CILP-2 secretion by acute injection.

To further understand CILP-2 physiology, we investigated CILP-2 expression in human and mouse tissues. Firstly, we found that CILP-2 mRNA and protein are more expressed in the muscle, fat, and liver in mice, suggesting that three insulin target tissues can secrete CILP-2 into circulation. In addition, we found that the mRNA expression of CILP-2 is the highest in muscle, followed by fat and liver. Therefore, we speculate that in these tissues, the muscle tissue secretes most CILP-2, followed by fat and liver. We recognized that these tissues might be important contributors to CILP-2 systemic levels. CILP-2 might be a novel myokine, adipokine or hepaticokine related to IR, like irisin, adiponectin, or fibroblast growth factor 21 (FGF21) (Yamauchi et al., 2001; Li et al., 2015; Liu et al., 2018). Although CILP-2 expression at mRNA levels in muscle tissues was significantly higher than that in adipose tissues, the CILP-2 expression at protein levels was similar. This can be explained by the following two reasons: first, muscle tissues may secrete more CILP-2 protein into the circulation compared with adipose tissue; second, CILP-2 may have the tissue-specific post-transcriptional modification.

In IR mice (including HFD-fed ADI-KO and db/db mice) and T2DM patients, the CILP-2 expression was significantly increased in muscle and liver but decreased in fat. This scenario is similar to some adipokines, such as adiponectin and Zinc-α2-glycoprotein (Sutinen, et al., 2003; Oana, et al., 2005; Barnea, et al., 2006; Yang, et al., 2013). We speculate that it may be due to (i) a compensatory down-regulation in adipose tissue to counteract the metabolic stress imposed by obesity; (ii) the decreased production of CILP-2 in adipose tissue may contribute to hepatic IR, similar to adiponectin (Sutinen, et al., 2003); (iii) IR may have a tissue-specific effect on CILP-2 expression (Barnea, et al., 2006). However, further detailed studies are needed to conclusively address these issues.

To understand the direct impact of CILP-2 on HGP and insulin sensitivity *in vivo*, EHCs were performed to examine the metabolic response of CILP-2 overexpression in IR state. We found that CILP-2 overexpression in both liver and muscle tissues further impaired glucose tolerance, reduced glucose utilization in peripheral tissues and blunted the ability of insulin to inhibit HGP *in vivo*. The changes in glucose output occurred at similar insulin levels and accounted for the effect of CILP-2 on whole-body glucose metabolism, suggesting its role in the mediation of hepatic and peripheral glucose homeostasis. Parallel to the results of *in vivo* study, the expression of PEPCK, a key gluconeogenic enzyme, was significantly increased in Ad-*CILP-2* treated hepatocytes, indicating that increased CILP-2 expression promotes gluconeogenesis and increases HGP. Therefore, we believe that hyperglycemia increases the expression and secretion of CILP-2, which in turn promote glyconeogenesis and lead to a vicious cycle to promote the progression of diabetes.

The insulin signaling pathway is activated when insulin binds to an InsR, resulting in phosphorylation of insulin receptor substrate-1 and consequent activation of phosphatidylinositol 3-kinase/PDK1/Akt (Saltiel and Kahn, 2001; Fang, et al. 2017). Therefore, we examined the effects of CILP-2 overexpression on phosphorylation of InsR and Akt, the two important molecules for insulin signal transmission (Saltiel and Kahn, 2001), in hepatocytes. Overexpression of CILP-2 leads to a decrease in phosphorylation of InsR and Akt in the hepatocyte. As seen in EHC study *in vivo*, the data from the study *in vitro* also indicate that CILP-2 overexpression blunts hepatic insulin signaling.

To understand the molecular mechanism that CILP-2 increases PEPCK expression, we demonstrated that CILP-2 positively regulated the transcriptional activity of PEPCK promoter. Furthermore, the detailed deletion analysis of PEPCK promoter revealed that the region of the PEPCK promoter responsible for CILP-2 activation was located within −217 bp to −57 bp upstream of the transcriptional start site. The results thus revealed an interaction between CILP-2 and PEPCK, by which HGP was regulated.

It has been reported that some hormones, such as insulin and glucocorticoids, regulated the transcriptional activity of the PEPCK gene (Lucas and Granner, 1992; Waltner-Law et al., 2003), while a set of DNA elements mediated a hormone response, which is termed hormone response units (HRU). Here, we found that DNA elements that comprise the PEPCK-CILP-2 response unit were located between positions −217 bp to −57 bp upstream of the transcriptional start site of the PEPCK promoter. We named it CILP-RU. Thus, we propose that CILP-2 could be a mediator for glucose homeostasis *in vivo*.

In summary, the current study provided the first evidence that CILP-2 is a novel secreted protein and demonstrated that circulating CILP-2 levels were elevated in both IGT and nT2DM subjects and associated with IR. CILP-2 overexpression promoted hepatic IR in HFD-fed mice, increased PEPCK expression and suppressed insulin signaling in hepatocytes. Thereby, we have identified a direct interaction between CILP-2 and PEPCK in the regulation of HGP.

## Material and methods

### Generation of CILP-2 expression vector

Sequence encoding FLAG tag was introduced to full-length CILP-2 cDNA fragment (3471 bp) and cloned into the *Bgl*II and *Eco*R sites of pIRES2-EGFP vector to generate CILP-2-F. To generate Ad-*CILP-2*, *CILP-2*-Flag were recombined into the Gateway-based pAd-AdEasy™ XL vector, according to the manufacturer’s instructions. The sequences from a mouse cDNA library (GI: 28913720) and encoding FLAG tag were listed in Supplementary Table S5. As a control, the recombinant adenovirus encoding enhanced green fluorescence protein (Ad-*GFP*; Clonetech) was prepared as described previously (Li et al., 2011a).

### The in vitro secretion studies

To determine whether CILP-2 is a secreted protein, we performed an *in vitro* study as previously described (Jia et al., 2014). Briefly, HEK-293T cells were transfected with CILP-2-F or an empty vector. Five milliliters of culture medium and one-third of the cell lysate from a 10-cm dish were immunoprecipitated with Flag antibody beads. Precipitates were separated by polyacrylamide gel electrophoresis, immunoblotted with CILP-2 antibody, and detected with enhanced chemiluminescence. A cell viability assay was performed using CCK-8 assay (Beyotime) according to the manufacturer’s instructions.

### Cross-sectional studies

A total of 310 subjects, 103 nT2DM patients (nT2DM group), 102 IGT subjects (IGT group), and 105 age-matched normal controls (NGT group), were recruited in this study. The diagnosis of IGT and T2DM was based on World Health Organization 1998 diagnostic criteria (Alberti and Zimmet, 1998). The individuals with T2DM or IGT had not been treated with any hypoglycemic agents and diet control. The patients with type 1 diabetes, macrovascular or microvascular complications, liver cirrhosis, congestive heart failure, or other major diseases were excluded. Normal controls were recruited from the individuals that underwent routine medical check-ups. Written voluntary consent was obtained from all subjects before their participation. This study was approved by the ethics committee of Chongqing Medical University in accordance with the World Medical Association Declaration of Helsinki (Clinical Trial Registration Number: ChiCTR-OCS-13003185).

### OGTT and EHC in humans

OGTT and EHC were performed on 30 healthy volunteers (15 women and 15 men) aged 18–35 years and with BMI 18–24 kg/m^2^ as previously described (Yang et al., 2013; Jia et al., 2014).

### Exenatide treatment

Thirty patients (15 men and 15 women; 58 ± 8 years) received subcutaneous Exenatide (5 μg) injection twice daily for 12 weeks. Inclusion criteria included age 40–70 years, BMI 25–35 kg/m^2^, and HbA1c 7%–8.5%. Blood samples for CILP-2 and biochemical measurements were obtained at 8 h prior treatment and on Day 2 of the last administration.

### Anthropometric, biochemical, and CILP-2 measurements

Plasma glucose, HbA1c, HOMA-IR, and HOMA of insulin secretion (HOMA-IS) were detected and calculated as previously reported (Albareda et al., 2000; Jia et al., 2014). Circulating CILP-2 concentrations were examined with an ELISA kit (Elisa Biotech Co., Ltd). The limit of detection was 6.25 ng/L, and intra- and interassay variations were <8% and <10%, respectively. The linear range of the assay was 25–1600 ng/L. The ELISA kit had been validated by the dealer, showing high sensitivity and excellent specificity for detection of human CILP-2, without significant cross-reactivity or interference.

### Tissue studies in human and mice

Gastrocnemius muscle, abdominal fat, and/or liver were obtained from 10 T2DM patients and 10 healthy subjects undergoing surgical procedures and also from mice. For each, 200 mg of tissues was mobilized and excised. Tissue samples were immediately frozen by liquid nitrogen and stored at −160°C until use.

### Animal preparation and EHC studies

All animal experiments were approved by the Ethics Review Committee for Animal Experimentation of Chongqing Medical University and adhered to ARRIVE guidelines. C57BL/6 J, db/db, and ADI KO mice were purchased from the animal center at Chongqing Medical University or Shanghai Biomodel Organism Sci & Tech Develop CO., Ltd, respectively. Eight-week-old db/db, AID KO, and C57BL/6 J (WT) male mice were fed either a standard chow diet (NC, 16% kcal from fat) or a high-fat diet (HFD, 45% kcal from fat; Medicine Inc. Jiangsu, China) for 12 weeks. For glucose tolerance tests (GTTs) and EHC study, male WT mice were injected with Ad-*CLIP-2* or Ad-*GFP* (1 × 10^9^ pfu in 100 μl of PBS) once a week via tail vein or bilateral gastrocnemius muscles (intramuscular injection) at weeks 11 and 12 of HFD feeding. Three days before GTT or EHC experiments, catheters were placed into the right internal jugular vein and left carotid artery. GTT and EHC experiments were performed as previously described (Sun et al., 2009; Wu et al., 2014; Xu et al., 2016; Tubbs et al., 2018).

### Cell culture and adenoviral-mediated gene overexpression

HepG2 cells were cultured in Dulbecco modified Eagle medium supplemented with 10% fetal bovine serum in 6-well plates (Li et al., 2011b). MPHs were isolated from the C57BL/6 J mice as previously described (Dong et al., 2004) and cultured in RPMI-1640 for 24 h. Cells were transfected with Ad-*CILP-2* or Ad-*GFP* followed by treatment with or without 100 nM insulin for 10 min (Li et al., 2010). CILP-2 protein expression and insulin signal molecules were examined by western blots.

### CILP-2 expression and secretion in HepG2 cells

HepG2 cells were cultured as described above, and treated with 5 mM or 30 mM glucose for 24 h. CILP-2 mRNA expression in cell lysates was measured by qRT-PCR. To examine CILP-2 secretion, the culture medium was collected and CILP-2 was measured using an ELISA kit.

### mRNA and protein analysis

qRT-PCR was performed for mRNA expression levels. The sequences for CILP-2 were shown in Supplementary Table S5. Protein analysis was performed with western blots as described previously (Jia et al., 2014). Primary antibodies included anti-CILP-2 (1:200 dilution, Abcam Inc.), anti-PEPCK, anti-InsR/phospho-InsR, anti-Akt/phospho-Akt (Cell Signaling Technology), and β-actin (Santa Cruz Inc.). For detecting CILP-2 protein in circulation, 500 μl human serum was incubated with polyclonal CILP-2 antibody or pre-immune IgG (control) and protein A-Sepharose beads and subjected to immunoblotting with polyclonal anti-CILP-2 antiserum (1:1000 dilution) as previously described (Jia et al., 2014).

### Plasmid construction and luciferase assay

The luciferase reporter plasmids pPEPCK (−2112/+391)-Luc and PEPCK promoter truncations were constructed by PCR amplification. The primers were given in Supplementary Table S6. The PCR amplification was carried out for 30 cycles. PCR products were digested with *Bgl*II and *Hin*dIII and inserted into pGL3-basic at the corresponding restriction sites. DNA sequencing was used to identify the sequence of all subcloned fragments.

For the dual-luciferase reporter assay, HEK-293 cells were grown in 24-well plates and co-transfected for 24 h with CILP-2-F or pIRES2-GFP (0.38 μg), 0.38 μg/well PEPCK-luciferase reporter plasmid, and 0.04 μg pRL-SV40 (Promega) by using Lipofect Amine^TM^ 2000 Reagent (Gibco BRL) according to the manufacturer’s instruction. The transfected cells were washed three times with phosphate-buffered saline and lysed with Passive Lysis Buffer. Luciferase assays were performed using the Dual- Luciferase Reporter Assay System (Promega).

### Statistical analysis

All analyses were performed by using PASW Statistical Package (version 20.0 SPSS Inc.). Data were expressed as mean ± SD or SEM. Comparison analyses between groups were carried out by using ANOVA, unpaired *t*-test or paired *t*-test. Normal distribution of the data was determined by Kolmogorox–Smirnov test. Some values were skewed and logarithmically transformed to obtain a normal distribution. Simple and multiple regression analyses were used to examine the association between circulating CILP-2 and the values of other biomarkers. The association of circulating CILP-2 with IGT and T2DM was examined by logistic regression analysis. *P*-values < 0.05 were thought as significant.

## Supplementary Material

mjz016_Supplementary_material
