# Supplementary material for: CILP-2 is a novel secreted protein and associated with insulin resistance
Source: J Mol Cell Biol. 2019 Jun 6;11(12):1083–94. doi: 10.1093/jmcb/mjz016 (PMC6934158; doi:10.1093/jmcb/mjz016)
Supplement: mjz016_Supplementary_material [file mjz016_supplementary_material.pdf]

CILP-2 is a Novel Secreted Protein and Associated with Insulin Resistance

Corresponding author

Gangyi Yang and Ling Li, Department of Endocrinology, the Second Affiliated Hospital,

Chongqing Medical University, 400014 Chongqing, China.

Tel: +86-23-68485216 / Fax: +86-23-68485005.

E-mail: gangyiyang@163.com and liling31@hotmail.com

Supplementary Table S1 Simple and multiple regression analyses of variables associated with circulating CILP-2 levels in the study population.

	Simple		Multiple	
	r	***P***	***b***	**P**
Age (yr)	0.025	0.662	----------	----------
BMI (kg/m^2)	0.041	0.482	----------	----------
WHR	0.341	< 0.001	0.827	< 0.001
FAT (%)	0.034	0.555	----------	----------
SBP (mmHg)	0.079	0.171	----------	----------
DBP (mmHg)	0.098	0.089	----------	-----------
TG (mmol/L)	0.265	< 0.001	----------	-----------
TC (mmol/L)	0.100	0.083	----------	-----------
HDL-C (mmol/L)	-0.138	< 0.05	----------	-----------
LDL-C (mmol/L)	0.011	0.851	----------	-----------
FBG (mmol/L)	0.202	< 0.001	----------	-----------
2h-BG (mmol/L)	0.296	< 0.001	----------	-----------
HbA1c (%)	0.150	< 0.01	----------	-----------
FFA (μmol/L)	0.102	0.079	----------	-----------
FIns (mU/L)	0.246	< 0.001	----------	-----------
2h-Ins (mU/L)	0.181	< 0.01	----------	-----------
HOMA-IR	0.396	< 0.001	0.033	< 0.001

CILP-2 and TG were skewed and logarithmically transformed to obtain a normal distribution. In multiple linear stepwise regression analysis, values included for analysis were age, BMI, WHR, FAT(%), SBP, DBP, TC, TG, HDL-C, LDL-C, FFA, FBG, 2h-BG, HbA1c(%), FIns, 2h-Ins and HOMA-IR. BMI, body mass index; WHR, waist-to-hip ratio; FAT (%), visceral fat percentage; SBP, systolic blood pressure; DBP, diastolic blood pressure; TG, triglyceride; TC, total cholesterol; HDL-C, high-density lipoprotein cholesterol; LDL-C, low-density lipoprotein cholesterol; FBG, fasting blood glucose; 2h-BG, 2-h blood glucose after glucose overload; FFA, free fatty acids; FIns, fasting insulin; 2h-Ins, 2-h plasma insulin after glucose overload; HOMA-IR, HOMA-insulin resistance index.

Supplementary Table S2 Association of circulating CILP-2 with IGT and T2DM in fully adjusted models.

Model adjust	IGT			T2DM		
	OR	95%CI	*P*	OR	95%CI	*P*
Age	1.015	1.007-1.022	<0.001	1.020	1.013-1.028	< 0.001
Age, Sex	1.015	1.007-1.022	< 0.001	1.020	1.013-1.028	< 0.001
Age, Sex, BMI	1.015	1.008-1.023	< 0.001	1.020	1.013-1.028	< 0.001
Age, Sex, BMI, FAT(%)	1.015	1.008-1.023	< 0.001	1.021	1.013-1.028	< 0.001
Age, Sex, BMI, FAT(%), WHR	1.015	1.007-1.022	< 0.001	1.020	1.013-1.028	< 0.001
Age, Sex, BMI, FAT(%), WHR SBP, DBP	1.015	1.007-1.022	< 0.001	1.020	1.012-1.028	< 0.001
Age, Sex, BMI, FAT(%), WHR SBP, DBP, Lipid profile	1.160	1.008-1.241	< 0.001	1.210	1.013-1.291	< 0.001

Results of binary logistic regression analysis are presented as the odds ratio (OR) of being in IGT and T2DM status with an increase in circulating CILP-2. IGT, impaired glucose tolerance; T2DM, type 2 diabetes mellitus; IR, insulin resistance; BMI, body mass index; WHR, waist-to-hip; FAT (%), visceral fat percentage; SBP, systolic blood pressure; DBP, diastolic blood pressure; CI, confidence interval; lipid profile, including TC, FFA, TG, LDL-C and HDL-C.

Supplementary Table S3 Clinical characteristics pre- and post-treatment with exenatide in T2DM patients.

Item	Pre-treatment	Post-treatment
BMI (kg/m^2)	24.6 ± 3.1	23.3 ± 2.7
WHR	0.89 ± 0.06	0.88 ± 0.06
FAT (%)	29.4 ± 5.5	28.6 ± 4.8
SBP (mmHg)	131.1 ± 10.1	129.0 ± 10.6
DBP (mmHg)	78.1 ± 8.0	79.4 ± 6.3
TG (mmol/L)	2.00 ± 1.02	1.87 ± 0.99
TC (mmol/L)	4.85 ± 1.08	5.05 ± 1.31
HDL-C(mmol/L)	1.30 ± 0.27	1.44 ± 0.49
LDL-C(mmol/L)	2.67 ± 0.94	2.87 ± 1.07
FFA (umol/L)	0.63 ± 0.21	0.53 ± 0.19*
FBG (mmol/L)	9.94 ± 2.27	8.08 ± 1.50**
PBG (mmol/L)	17.2 ± 4.4	14.0 ± 4.9*
FIns (mU/L)	13.8 ± 12.7	14.0 ± 16.2
HOMA-IR	6.12 ± 5.71	5.02 ± 6.08*

BMI, body mass index; WHR, waist-to-hip ratio; FAT (%), visceral fat percentage; SBP, systolic blood pressure; DBP, diastolic blood pressure; TG, triglyceride; TC, total cholesterol; HDL-C, high-density lipoprotein cholesterol; LDL-C, low-density lipoprotein cholesterol; FFA, free fatty acids; FBG, fasting blood glucose; PBG, postprandial plasma glucose; FIns, fasting insulin; HOMA-IR, HOMA-insulin resistance index. Values were given as mean ± SD (n=30). *P <0.05, **P <0.01 compared with pre-treatment.

Supplementary Table S4 Clinical features of T2DM patients with CILP-2 expression analysis.

**Variable**	**Controls (n=10)**	**T2DM (n=10)**
Age (year)	56.9 ± 8.2	58.2 ± 8.0
BMI (kg/m^2)	23.6 ±1.6	24.6 ± 2.3
WHR	0.84 ± 0.06	0.89 ± 0.08*
FAT (%)	29.8 ± 6.8	33.0 ± 5.5
SBP (mmHg)	118.4 ± 8.4	130.6 ± 11.4*
DBP (mmHg)	77.4 ± 5.7	80.1 ± 5.67
TG (mmol/L)	1.21 ± 0.41	1.92 ± 0.31**
TC (mmol/L)	4.64 ± 0.59	4.85 ± 0.49
HDL-C (mmol/L)	1.35 ± 0.44	1.24 ± 0.36
LDL-C (mmol/L)	2.58 ± 0.39	2.63 ± 0.39
FBG (mmol/L)	5.37 ± 0.33	9.16 ± 2.09**
PBG (mmol/L)	6.74 ± 0.91	15.3 ± 3.6**
FIns (mU/L)	8.16 ± 4.12	10.1 ± 5.5
HOMA-IR	1.92 ± 1.31	4.25 ± 1.78**
HbA1c	5.71 ± 0.32	7.89 ± 0.42**

BMI, body mass index; WHR, waist-to-hip ratio; FAT(%), visceral fat percentage; SBP, systolic blood pressure; DBP, diastolic blood pressure; TG, triglyceride; TC, total cholesterol; HDL-C, high-density lipoprotein cholesterol; LDL-C, low-density lipoprotein cholesterol; FBG, fasting blood glucose; PBG, postprandial plasma glucose; FIns, fasting insulin; HOMA-IR, HOMA-insulin resistance index. Values were given as mean ± SD. *P <0.05, **P <0.01 compared with controls.

Supplementary Table S5 Characteristics of the specific primers used for RT-PCR analysis.

Gene	Forward and reverse primers
β-actin	Forward : 5'-GCTGTCCCTGTATGCCTCT-3'
	Reverse : 5'-GATGTCACGCACGATTTCC-3'
CILP-2 (human)	Forward : 5'-GGATGTGAGCCGCCGTGA-3'
	Reverse : 5'-CAGCCGTCCCTGTCACCCTC-3'
CILP-2 (mouse)	Forward : 5'-GAATTCATGAAGAGTAAAAAGCCCCTG-3'
	Reverse: 5'-GTCGACCTACAGCAG GACCTTGGTG-3'
FLAG	GATTACAAGGACGACGATGACAAG

Supplementary Table S6 Primers used for PEPCK promoter recombinant plasmids.

Promoter regions		Primers
-2112/+391	F	5′-GGAAGATCTGAATCATTACCGTCCTTCCA-3′
	R	5′-CCCAAGCTTCCAGTTGTCATACTTCTTCAG-3′
-1257/+391	F	5′-GGAAGATCTTCACCCTGTGTTCTCCAAAC-3′
	R	5′-CCCAAGCTTCCAGTTGTCATACTTCTTCAG-3′
-1097/+391	F	5′-GGAAGATCTAACTCTAAGACTTGCCAGG-3′
	R	5′-CCCAAGCTTCCAGTTGTCATACTTCTTCAG-3′
-857/+391	F	5′-GGAAGATCTTGTTCATAGCCTCCACCTCAG-3′
	R	5′-CCCAAGCTTCCAGTTGTCATACTTCTTCAG-3′
-457/+391	F	5′-GGAAGATCTTGACCCACCTGCCTGTTAAG-3′
	R	5′-CCCAAGCTTCCAGTTGTCATACTTCTTCAG-3′
-217/+391	F	5′-GGAAGATCTTGGCGAGCCTCCCTGGGTG-3′
	R	5′-CCCAAGCTTCCAGTTGTCATACTTCTTCAG-3′
-57/+391	F	5′-GGAAGATCTTCCAAAGAGAAGAAAGGTAAG-3′
	R	5′-CCCAAGCTTCCAGTTGTCATACTTCTTCAG-3′

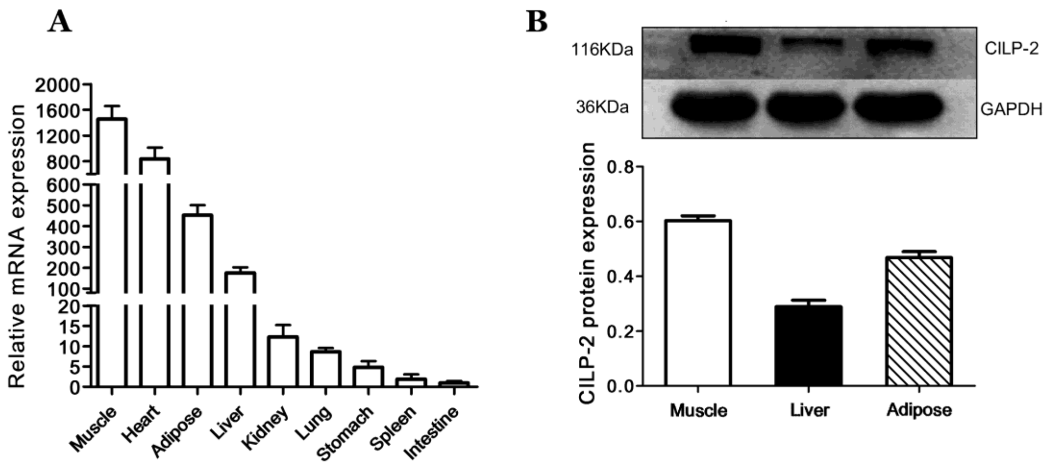

Supplementary Figure S1 Tissue distribution of CILP-2 expression in mice. **(A)** CILP-2 mRNA expression levels in different tissues. **(B)** Protein expression in muscle, liver and adipose tissues. Values are mean ± SEM (n=3).

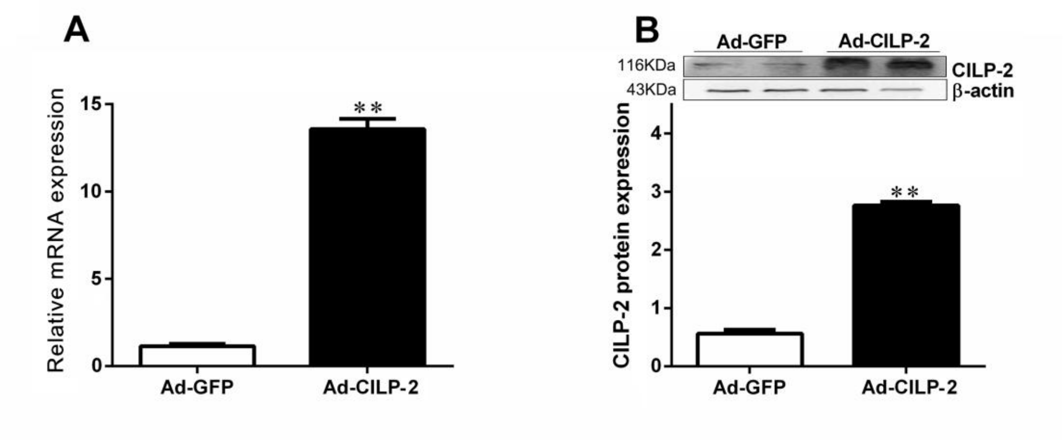

Supplementary Figure S2 CILP-2 expression in the liver of mice. Male C57BL/6J mice were injected with Ad-*CILP-2* or Ad-*GFP via* tail vein and sacrificed 10 days later. The expression of CILP-2 mRNA **(A)** and protein **(B)** in the liver. Values are mean ± SEM (n=3). ***P* <0.01 *vs.* Ad-*GFP*.

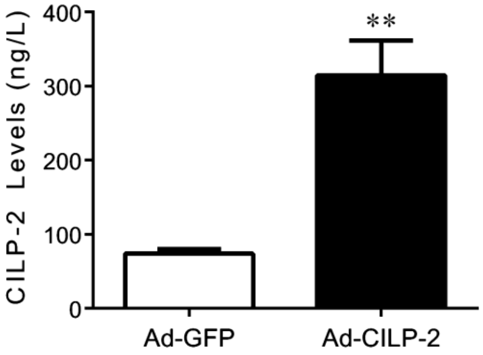

Supplementary Figure S3 Circulating CILP-2 levels in mice. Male C57BL/6J mice were injected with Ad-*CILP-2* or Ad-*GFP via* tail vein and sacrificed 10 days later. Circulating CILP-2 levels were measured by ELISA. Values are mean ± SEM (n=3). ***P* <0.01 *vs.* Ad-*GFP*.

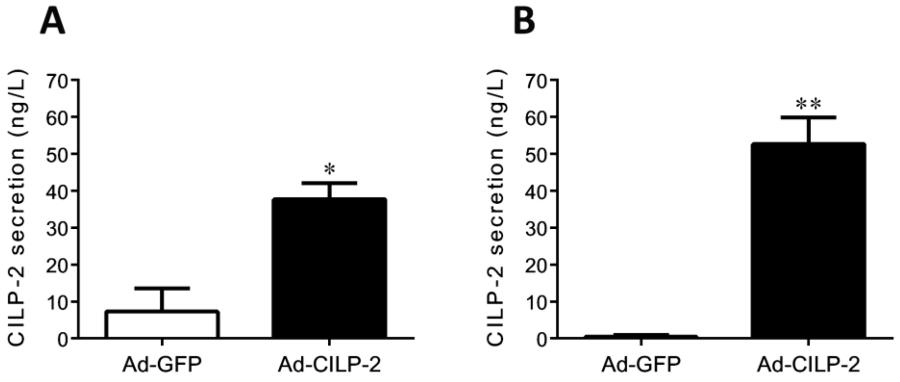

Supplementary Figure S4 CILP-2 protein secretion in cell medium. HepG2 cells **(A)** and MPHs **(B)** were transfected with Ad-*CILP-2* or Ad-*GFP*. The CILP-2 contents in cell medium were determined by an ELISA kit. MPHs, mouse primary hepatocytes. Values are mean ± SEM (n=3). **P* < 0.05, ***P* < 0.01 *vs.* Ad-*GFP*.

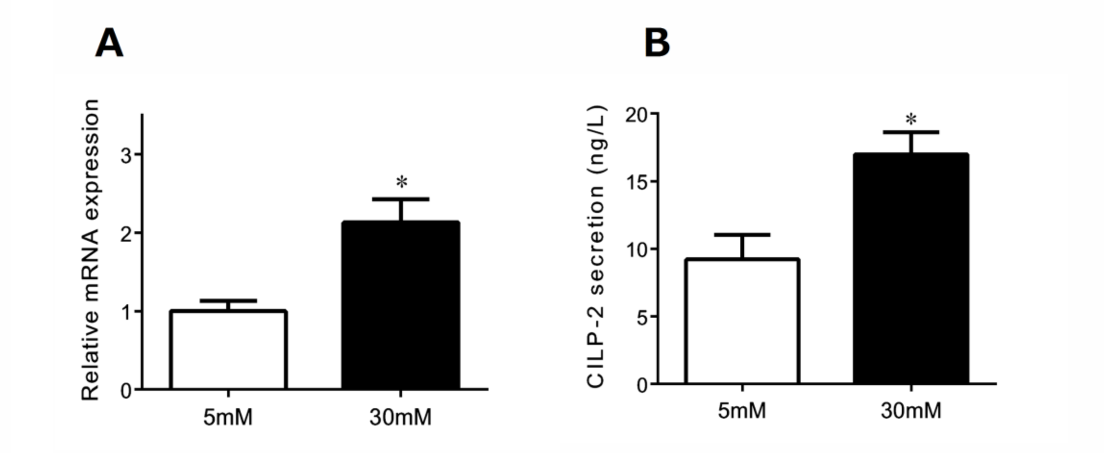

Supplementary Figure S5 The effects of high glucose on CILP-2 mRNA expression and protein secretion in HepG2 cells. HepG2 cells were cultured as indicated in Methods and treated with 5 mM or 30 mM glucose for 24 h. **(A)** CILP-2 mRNA expression in cell lysates. **(B)** The protein concentrations of CILP-2 in the conditioned medium. Values are mean ± SEM (n=3). *$P < 0.05$ *vs.* 5 mM.
